# Sustainable Control of Onchocerciasis: Ocular Pathology in Onchocerciasis Patients Treated Annually with Ivermectin for 23 Years: A Cohort Study

**DOI:** 10.1371/journal.pone.0098411

**Published:** 2014-06-02

**Authors:** Méba Banla, Solim Tchalim, Potochoziou K. Karabou, Richard G. Gantin, Aide I. Agba, Abiba Kére-Banla, Gertrud Helling-Giese, Christoph Heuschkel, Hartwig Schulz-Key, Peter T. Soboslay

**Affiliations:** 1 Onchocerciasis Reference Laboratory, Institut National d'Hygiène, Sokodé, Togo; 2 Centre Hospitalier Universitaire Campus, Université de Lomé, Lomé, Togo; 3 National Onchocerciasis Control Programme, Kara, Togo; 4 Institute for Tropical Medicine, University Clinics of Tübingen, Tübingen, Germany; Kenya Medical Research Institute (KEMRI), Kenya

## Abstract

The evolution and persistence of ocular pathology was assessed in a cohort of *Onchocerca volvulus* infected patients treated annually with ivermectin for 23 years. Patients were resident in rural Central and Kara Region of Togo and ocular examinations included testing of visual acuity, slit lamp examination of the anterior eye segment and the eye fundus by ophthalmoscopy. Before ivermectin treatment, vivid *O.volvulus* microfilariae (MF) were observed in the right and left anterior eye chamber in 52% and 42% of patients (n = 82), and dead MF were seen in the right and left cornea in 24% and 15% of cases, respectively. At 23 years post initial treatment (PIT), none of the patients (n = 82) presented with MF in the anterior chamber and cornea. A complete resolution of punctate keratitis (PK) lesions without observable corneal scars was present at 23 years PIT (p<0.0001), and sclerosing keratitits (SK) lessened by half, but mainly in patients with lesions at early stage of evolution. Early-stage iridocyclitis diminished from 42%(rE) and 40%(lE) to 13% (rE+lE)(p<0.0001), but advanced iridocyclitis augmented (p<0.001) at 23 years PIT compared to before ivermectin. Advanced-stage papillitis and chorioretinitis did not regress, while early-stage papillitis present in 28%(rE) and 27%(lE) of patients at before ivermectin regressed to 17%(rE) and 18%(lE), and early-stage chorioretinitis present in 51%(rE+lE) of cases at before ivermectin was observed in 12%(rE) and 13%(lE) at 23 years PIT (p<0.0001). Thus, regular annual ivermectin treatment eliminated and prevented the migration of *O. volvulus* microfilariae into the anterior eye chamber and cornea; keratitis punctata lesions resolved completely and early-stage sclerosing keratitits and iridocyclitis regressed, whilst advanced lesions of the anterior and posterior eye segment remained progressive. In conclusion, annual ivermectin treatments may prevent the emergence of ocular pathology in those populations still exposed to *O.volvulus* infection.

*Trial Registration*: www.pactr.org PACTR201303000464219)

## Introduction

In West Africa, onchocerciasis is successfully controlled by the African Program for Onchocerciasis Control (APOC) and in large parts the disease is no longer considered a public health problem [1], and in certain onchocerciasis endemic foci in Mali and Senegal elimination is considered achievable [2], [3]. Complete control or elimination of onchocerciasis in the near future may be attainable with high rates of ivermectin treatment coverage (>85%) administered twice annually, and applied for more than 15 years which is expected to exceed the reproductive life span and natural life expectancy of adult *Onchocerca volvulus* [1], [4]. However, onchocerciasis is considered as not eradicable in Africa using the currently available tools [5]. Ivermectin treatment will reduce within a few days *O. volvulus* microfilaria (MF) numbers in patients' skin by more than 70% [6], [7], and progressively during the following months MF will decline in the anterior chamber and cornea of the patients' eyes [8], [9], but until 12 month post initial treatment (PIT) MF numbers may increase again [10], [11]. Ivermectin therapy is associated with mild systemic adverse responses [6] and repeated treatments will alleviate hyper-reactive skin manifestations in patients and prevent the progression to chronic dermal pathology [12], [13]. In onchocerciasis endemic foci in Cameroon and Nigeria, recent works have shown that community directed treatment with ivermectin (CDTI) will not interrupted *O. volvulus* transmission [13], and CDTI did not prevent evolution of ocular onchocerciasis [14], [15], respectively. Despite repeated ivermectin treatments for years female adult *O. volvulus* remain reproductive [16], and unresponsiveness of *O. volvulus* to ivermectin treatment in some areas of West Africa might have emerged as a further problem for onchocerciasis control [17]. Replicated longitudinal surveys are required to determine the impact of ivermectin on dermal and ocular pathology in the APOC area, notably in the risk zones of the former Onchocerciasis Control Programme (OCP) where low level parasite transmission and few active *O. volvulus* infections still persist [18]; here the risk for recurrence of onchocerciasis may threaten the success of disease control.

The present ophthalmological study examined the evolution and changes of ocular pathology in a cohort of onchocerciasis patients permanently resident in central Togo and treated annually with ivermectin for 23 years. Patients were initially enrolled in a phase III double-blind placebo-controlled dose finding study of ivermectin, and from month 18 post initial treatment (PIT) onwards all patients received annual ivermectin treatment with 150 ug/kg as a single dose. Ocular onchocerciasis manifestations in ivermectin treatment groups evolved, persisted or regressed similarly in the patient groups [7], and ivermectin treatments applied annually for more than 2 decades permanently eliminated ocular *O.volvulus* microfilaria, and such regular interventions may prevent the emergence of ocular pathology in those populations still exposed to *O.volvulus* infection.

## Methods

### Ethics Statement

The protocol of the study was reviewed and approved by the Advisory Council of the Ministry of Health in Togo, the Committee on Research Involving Human subjects of the World Health Organization Ethics Commission and the Medical Board at University Clinics of Tübingen. The study has been registered at the Pan African Clinical Trial Registry and the WHO clinical trial registry platform (PACTR201303000464219: Onchocerciasis Ocular Pathology post Ivermectin; date of registry: 25/11/2012). Authorization and approval granted by the Ministry of Health in Togo were re-approved every 4–5 years (no. 2824/87/MSP-ASCF; no. 292//MS/CAB; no. 261//MSP/ DGSP/DRSP-RC, no. 0407/2007/MS/CAB/DGS, no. 0129/2011/MS/CAB/DGS/ DPLET/CBRS). The protocol for this trial and supporting CONSORT checklist are available as supporting information; see Checklist S1 and Protocol S1. At beginning (in 1985), this study was a phase III double-blind placebo-controlled dose finding study of ivermectin for treatment of onchocerciasis. The aims of the work, risks, procedures of examination and follow up intervals were explained thoroughly to the respective village population, the village authorities and honourables, notably the village chief council. Before the very beginning of the phase III double-blind placebo-controlled dose finding study of ivermectin, all patients gave their consent for participation. The consent from each study participant was documented and confirmed by signature in the study protocol by the responsible physicians and/or their medical assistants. Consent for study participation by those less than 18 years of age was given verbally by each participant, and approval for participation was always obtained by the parents or the accompanying responsible adults. Written consent was not obligatory requested and such accepted by the national Togolese Health Authorities, i.e. the Ministry of Health, and approval for participation for those less than 18 years of age was always recorded by the responsible physician of the study in the study protocol. In 1985, the beginning of the phase III double-blind placebo-controlled dose finding study of ivermectin, the responsible authority for ethical approval of clinical trials was the Ministry of Health in Togo, which has approved this protocol. Until 54 months post initial treatment (PIT), and at each time patients were examined, patients' consent, absence or refusals were documented in the study protocol. For correct and complete understanding explanations were always given in the local language. Before each follow up survey, approval was obtained from the appropriate regional (Direction Regional de la Santé de la Population) and district-level (Direction Prefectural de la Santé) health authorities. At each follow up examination, all participants gave their informed oral consent; as such it was approved by the Review Boards owing to the low risk and routine nature of the ophthalmological examination procedures. Each patient's participation, absence or refusal was recorded in the individual study protocol sheets (MSD-Nr519: Ivermectin (MK933) versus placebo in onchocerciasis ophthalmologic examinations (OE).

### Patients

Patients were enrolled in the phase III double-blind placebo-controlled dose finding study of ivermectin for treatment of onchocerciasis. All patients from this study originated from villages in the Central and Kara Region of Togo, where *O. volvulus* infection was hyper- to meso-endemic. Patients were resident in the villages Bougabou, Bouzalo, Kemeni, Mo, Tabalo, Tchapossi, Sagbadai and Sirka. Patients were apparently healthy males and non-pregnant women with a body weight over 30 kg, without history of multiple allergies or drug intolerance, with normal laboratory parameters except for the clinical features of onchocerciasis, with skin biopsies positive for microfilaria of *O. volvulus* and with palpable nodules (onchocercomata). Criteria for exclusion were allergies or drug intolerance, concomitant infection with *Loa loa* or *Wuchereria bancrofti*, haematocrit below 30%, renal or hepatic disease, convulsions or other central nervous system disease, clinical signs suggestive of meningitis, pregnant females or nursing mothers and patients who had received microfilaricidal drugs during the preceding year. The protocol of the study was reviewed and approved by the Ethics Commission of the Medical Board at University of Tübingen, the Advisory Council of the Ministry of Health in Togo and the Committee on Research Involving Human subjects of the World Health Organization.

### Patient groups and examination during and after chemotherapy

In patients, the densities of microfilaria of *O. volvulus* (MF/mg) in the skin were determined at the right and left iliac crest and the right and left calf by means of corneascleral punch (Holth- or Walser-type) before ivermectin treatment and at day 3, at month 3, month 6 and at 1 year post treatment. Skin biopsies were immediately weighed and then immersed in 0.1 ml physiological saline in a well in a flat bottomed micro titre plate, and stored at room temperature in a high-humidity atmosphere. Microfilariae were counted after overnight incubation. Patients were randomly assigned to receive either 0(placebo), 100, 150, or 200 µg/kg of ivermectin. Patients were examined physically and intensity of signs and symptoms following ivermectin or placebo were scored, as described in detail previously by Greene and co-workers [5]. All onchocerciasis patients participated in regular surveys conducted by the Onchocerciasis Reference Laboratory (ORL) and the National Onchocerciasis Control Program (NOCP). Clinical data of all patients were gathered in a double-blind fashion, those of the ivermectin and placebo patients during the first 12 months after therapy. From months 18 post initial treatment (PIT) onwards all patients received annual ivermectin treatment (150 µg/kg) as a single dose distributed by the ORL and the NOCP in Togo. At regular intervals thorough physical, parasitological and ophthalmological examinations were conducted and the density of *O. volvulus* microfilariae (MF) was determined in skin biopsies (MF/mg skin) taken from the right and left hip.

### Ophthalmologic Examinations

#### Visual Acuity

The ocular examinations included the testing of visual acuity eye by eye with an illiterate E chart (SNELEN) placed 6 meters away from the patient's seat, and visual acuity was graded according to WHO/OCP criteria; blind were those with a visual acuity on one or both eyes of less than 1/20 (3/60 or unable to count fingers at 3 meters); impaired vision had those with a visual acuity on one or both eyes between 1/20 and less than 3/10 (6/18) and good vision had those with a visual acuity equal or greater than 3/10 (6/18). Note: Pin hole was used when visual acuity was less than 3/10 (6/18).

#### Slit Lamp Examination

The ophthalmology examinations until 36 months PIT were conducted by M.Banla together with other ophthalmologists, and thereafter, all follow-up examinations were conducted by one examiner (M. Banla). The anterior chamber and cornea were examined with a HAAG STREIT Slit Lamp 900 after the patients were placed in the head down position for at least two minutes. The microfilaria in the cornea and the anterior chamber of each eye were counted. Limbitis was diagnosed by the presence of limbal vessel dilatation, limbal oedema, and white globular opacities, and each sign was graded as absent, mild, moderate, or severe. Iridocyclitis in evolution was diagnosed by the presence of anterior chamber cells and flare with or without iris atrophy, anterior or posterior synechies and secondary cataract due to iridocyclitis. The classification of iridocyclitis was according to the criteria applied by the Onchocerciasis Control Program (OCP) iridocyclitis was graded as: absent, early stage iridocyclitis was without synechies and or secondary cataract. Advanced iridocyclitis was with synechies and/or secondary cataract. Sclerosing keratitis was graded according to the presence of limbal haze, the extent of characteristic corneal involvement starting in the nasal and or temporal periphery, being confluent inferiorly, or covering the pupil.

#### Posterior Eye Segment Examinations

Examinations until 36 months PIT were conducted by M.Banla together with other ophthalmologists, and thereafter, all follow-up examinations were conducted by one examiner (M. Banla). The fundus was examined by direct and indirect ophthalmoscopy after pupil dilatation with 1% tropicamide and 10% epinephrine hydrochloride. The classification of lesions was according to the criteria applied by the Onchocerciasis Control Program (OCP). Early stage chorioretinitis due to onchocerciasis was with patchy non confluent atrophies of the retinal pigmented epithelium (RPE). Advanced chorioretinitis due to onchocerciasis were at least confluent atrophies of the RPE of any grade alone or associated with other lesions as choroïdo retinitis atrophy of any grade or sub retinal fibrosis. Early stage papillitis due to onchocerciasis was patchy papillary bleeding with pale, atrophic papilla or papilla abnormally pink associated with other ocular onchocerciasis lesions. Advanced stage papillitis was post neuritis optical atrophy with vessel sheathing, with or without papillary or peri-papillary haemorrhages associated with other ocular onchocerciasis lesions. The presence and extent of intraretinal deposits, intraretinal pigment hypertrophy as well as any other abnormalities were noted. After initial treatment the ocular examinations were repeated at day 3 PIT and at three, six, and twelve and 48 months PIT. Thereafter, the physical, parasitological and ocular examinations were repeated in 3-5 year intervals by the ORL and NOCP.

### Statistical data analysis

JMP software (versions 9.0.0; SAS Institute) was used for statistical analysis of data. Due to multiple comparisons, the level of significance was adjusted according to Bonferroni–Holm (alpha  = 0.0025). The manifestations of ocular pathologies at different time points post treatment and between groups were evaluated by contingency analyses using Pearson's chi-square test, and unpaired data of patients were compared using Wilcoxon's rank sum test. Multivariate correlation analysis was applied to show relationships between pairs of variables, e.g. patients' age, treatment groups, Years post treatment, ocular manifestation of onchocerciasis.

## Results

### *O. volvulus* skin microfilaria in patients post ivermectin treatments

**Figure 1:**
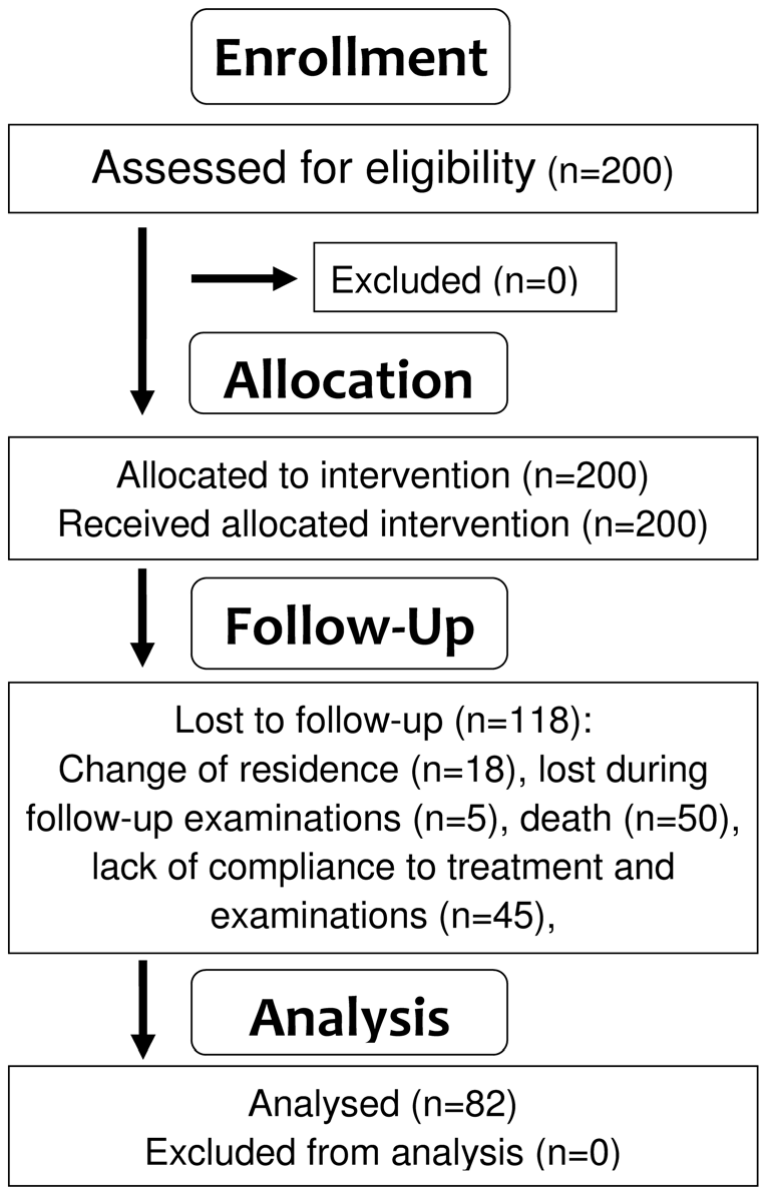
Flowchart showing the recruitment, allocation and follow up of study participants.

From the two hundred patients initially included in this longitudinal study, eighty two patients attended all follow-up examinations (Fig. 1). At 23 years post initial ivermectin treatment (PIT), the patient cohort consisted of 76 males and 5 females with a mean age of 54.3 years (min 36, max 77) (Table 1). Patients were initially enrolled in a phase III double-blind placebo-controlled dose finding study of ivermectin and were treated either with 100, 150 or 200 ug/kg ivermectin or received placebo; from months 18 post initial treatment (PIT) onwards, all patients received annual ivermectin treatment with 150 µg/kg as a single dose. In the ivermectin-treated patients' cohort, the density of *O. volvulus* microfilariae (MF) per mg skin (before: mean 73 MF/mg) decreased by 85% (11 MF/mg), 95% (4 MF/mg), 97% (3 MF/mg), 89% (8 MF/mg) and 59% (30 MF/mg) on day 3, month 3, month 6, month 12 and month 18 PIT, respectively. In the initial placebo group patients, the density of mf was reduced following ivermectin treatment by 75% to 17 MF/mg at 22 months PIT. At 4 years PIT, the mean mf density in n = 82 patients was 17 MF/mg and following ivermectin treatment it lessened to 2 Mf/mg at 3 days later. Thereafter, ivermectin treatments (150 µg/kg) were applied annually by the National Program for Onchocerciasis Control (PNLO/APOC) i.e. by community-directed ivermectin distribution. At 7 years PIT, all onchocerciasis patients were negative for mf in skin biopsies, and remained such during all the following re-examinations.

**Table 1:** Onchocerciasis patients' age and their profession at 23 years post initial ivermectin treatment.

Patients		n
Age (in groups)	35 y–45 y	20
	46 y–55 y	28
	56 y–65 y	19
	>65 y	15
Profession	Farmer	65
	House wife	5
	Administrator/Teacher	6
	Others (Trader, Retired)	6

### Visual Acuity

Despite annually repeated ivermectin treatments the visual acuity degraded gradually in the patients' cohort (Table 2). At the beginning of study, none of the patients presented with bilateral blindness. Good vision, as present in 88% of the patients at study begin, was observable only in 59% of the patients at 23 years PIT. At 23 years PIT, 17% of the treated patients were classified as bilaterally blind (Table 3). Noteworthy, this degradation cannot unequivocally be attributed to onchocerciasis as other ocular anomalies (Table 4), i.e. vascular retinopathy (18%), pterigium conjunctivae (20%) and band-shaped keratopathy (7%), emerged in the onchocerciasis patients cohort.

**Table 2:** Visual acuity in the onchocerciasis patients' cohort (n = 82) before ivermectin treatment, and at 4 years and 23 years post initial treatment (PIT).

	before ivermectin*(in %)			4 years PIT*(in %)			23 years PIT*(in %)		
Visual acuity	RE	LE	RLE	RE	LE	RLE	RE	LE	RLE
<1/20	2.4	2.4	0	4.9	7.3	1.2	2.4	7.3	17.1
≥1/20 and <3/10	2.4	1.2	6.1	1.2	2.4	9.7	10.9	3.6	11.7
≥3/10	3.6	4.9	87.7	7.5	3.6	80.4	1.2	4.9	58.5

For visual acuity an illiterate E chart (SNELEN) was used placed 6 meters away from the patient's seat. The patients' visual acuity was graded according to WHO criteria; blind were those with a visual acuity on the right (RE) or left eye (LE) or both eyes (RLE) of less than 1/20 (3/60 or unable to count fingers at 3 meters); impaired vision had those with a visual acuity between 1/20 (3/60) and less than 3/10 (6/18); good vision had those with a visual acuity equal or greater than 3/10. *Note: At before treatment and at 4 and 23 years PIT, visual acuity was not possible to determine in 11% (n = 9), 13% (11) and 17% (19) of the patients due to lack of comprehension, respectively.

**Table 3:** Causes of bilateral visual impairment and blindness in the onchocerciasis patients' cohort (n = 82) before ivermectin treatment, and at 4 years and 23 years post initial treatment (PIT).

Bilateral visual impairment and blindness	before ivermectin	4 years PIT	23 years PIT
Visual Acuity: ≥1/20 and <3/10	n (%)	n (%)	n (%)
Impaired by non onchocerciasis cataract	2 (2.4)	1 (1.2)	2 (2.4)
Impaired by onchocerciasis	3 (3.6)	4 (4.8)	5 (6.0)
Impaired by glaucoma	0	0	2 (2.4)
Impaired by other causes	0	3 (3.6)	1 (1.2)
**Total with impaired vision**	**5 (6.0)**	**8 (9.6)**	**10 (12.0)**
Visual Acuity: (<1/20 or <3/60)			
Blindness by non onchocerciasis cataract	0	1 (1.2)	2 (2.4)
Blindness by onchocerciasis	0	0	6 (7.3)
Blindness by onchocerciasis & other causes	0	0	3 (3.6)
Blindness by glaucoma	0	0	1 (1.2)
Blindness by other causes	0	0	2 (2.4)
**Total of blindness**	**0**	**1 (1.2)**	**14 (16.9)**

The patients' visual acuity was graded according to WHO criteria; blind were those with a visual acuity on the right (RE) or left eye (LE) or both eyes (RLE) of less than 1/20 (3/60 or unable to count fingers at 3 meters); impaired vision had those with a visual acuity between 1/20 (3/60) and less than 3/10 (6/18); good vision had those with a visual acuity equal or greater than 3/10.

**Table 4:** Ocular anomalies not caused by *Onchocerca volvulus* infection in patients (n = 82) examined before ivermectin treatment, and at 4 and 23 years post initial ivermectin treatment (PIT).

	before ivermectin (in %)	4 years PIT (in %)	23 years PIT (in %)
Band-shaped keratopathy	6.1	7.3	7.3
Glaucoma	4.9	4.9	4.9
Vascular retinopathy	2.4	4.9	18.2
Pterygium conjunctivae	2.4	4.8	19.5
Suspected glaucoma by Cup/disc ≥0.5	2.4	2.4	4.9
POA (primary optic atrophy)	1.2	1.2	1.2

### Microfilaria and lesions of the anterior eye segment

At the beginning of study, more than half of the onchocerciasis patients had living MF in their eyes' anterior chamber (AC) (Table 5). Living MF of *O.volvulus* in the AC (MFAC) regressed considerably in the patients from 52%(rE) and 42%(lE) at before ivermectin to 29%(rE) and 19%(lE) at 4 years and to 0% (rE+lE) at 23 years PIT, respectively. Similarly, dead MF of *O.volvulus* in the cornea (DMFC) diminished from 24%/15% (rE/lE) at before to 10%/7% (rE/lE) at 4 years PIT and to 0% (rE+lE) at 23 years PIT. Thus, regular annual treatment with ivermectin has achieved a complete elimination of MF from the AC and cornea of the patients' eyes.

**Table 5:** Microfilaria in the anterior chamber of the eye, the cornea, and puctate and sclerosing keratitis in onchocerciasis patients (n = 82) post ivermectin treatment. Differences were evaluated using Pearson's chi-square test.

	right EYE			left EYE		
	before ivermectin	4 years PIT$	23 years PIT	before ivermectin	4 years PIT	23 years PIT
**Microfilaria (MF) in the anterior chamber of the eye (MFAC)**						
MF Absent (in %)	37 (45)	54 (66)	78 (95)**	47 (57)	64 (78)	80 (98)**
Impossible to examine (in %)	2 (2)	4 (5)	4 (5)	1 (1)	2 (2)	2 (2)
MFAC >20 (in %)	1 (1)	1 (1)	0	1 (1)	1 (1)	0
MFAC 1–10 (in %)	40 (49)	20 (24)	0***	30 (37)	14 (17)	0***
MFAC 11–20 (in %)	2 (2)	3 (4)	0	3 (4)	1 (1)	0
**Dead microfilaria (MF) in the cornea (DMFC)**						
MF Absent (in %)	60 (73)	71 (87)	79 (96)	69 (84)	74 (90)	80 (98)
Impossible to examine (in %)	2 (2)	3 (4)	3 (4)	1 (1)	2 (2)	2 (2)
DMFC 1–10 (in %)	20 (24)	8 (10)	0**	12 (15)	6 (7)	0**
**Punctate Keratitis**						
Absent (in %)	44 (54)	20 (24)	80 (98)***	47(57)	26 (32)	80 (98)***
Impossible to examine (in %)	1(1)	2 (2)	2 (2)	1 (1)	1 (1)	2 (2)
Number of Lesions 1–10 (in %)	36 (44)	59 (72)	0***	33 (40)	50 (61)	0***
Number of Lesions 11–20 (%)	1 (1)	1 (1)	0	1 (1)	5 (6)	0
**Sclerosing Keratitis (SK)**						
Absent (in %)	74 (90)	58 (71)	75 (92)	73 (89)	60 (73)	77(94)
Impossible to examine (in %)	0	2 (2)	2 (2)	0	1 (1)	2 (2)
SK advanced (in %)	2 (2)	1 (1)	1 (1)	1 (1)	0	0
SK in evolution (in %)	6 (7)	21 (26)	4 (5)*	8 (10)	21 (26)	3 (4)*

$ PIT =  post initial treatment.

*p<0.001 compared to 4 years PIT;

**p<0.001 compared to before ivermectin;

***p<0.0001 compared to before ivermectin and 4 years PIT.

While MFAC and DMFC diminished by half in patients until 4 years PIT, the number of puctate keratitis (PK) lesions did not regress at 4 years PIT (Table 5). At 23 years PIT, PK lesions were resolved completely without leaving residual corneal scars. Sclerosing keratitis (SK) was present before ivermectin in 9%/11% (rE/lE), at 4 years PIT in 27%/26% (rE/lE) and at 23 years PIT, in 5%/4% (rE/lE) of the patients. The early evolving SK regressed in 80% of the patients, whilst the advanced-stage SK lesions did not resolve until 23 years PIT; only in one patient advanced SK reduced to early stage.

Iridocyclitis early-stage lesions regressed slightly from 42%/40% (rE/lE) to 26%/23% at 4 years PIT, but advanced stage lesions were detected more often (Table 6). At 23 years PIT, iridocyclitis early lesions were observed in 13% (rE+lE) with none occurring to be in evolution, advanced iridocyclitis lesions were present in 9%/10% (rE/lE) and none appeared as progressive. Thus, anterior eye segment lesions in an early stage of evolution disappeared completely, i.e. MFAC, DMFC and PK, and early-stage iridocyclitis diminished by 67% and 68%, but advanced-stage iridocyclitis as observed in 1–2% of the patients at before was present in 9–10% at 23 years PIT. Notably, cataract (44%) emerged in the onchocerciasis patients' cohort (Table 6).

**Table 6:** Iridocyclitis, cataract, papillitis and chorioretinitis in onchocerciasis patients (n = 82) post ivermectin treatment. Differences were evaluated using Pearson's chi-square test.

	right EYE			left EYE		
	before ivermectin	4 years PIT$	23 years PIT	before ivermectin	4 years PIT	23 years PIT
**Iridocyclitis**						
1/2 Mydriase (in %)	1 (1)	1(1)	1 (1)	1 (1)	1 (1)	1 (1)
Iris Normal (in %)	36 (44)	45 (55)	63 (77)**	34 (42)	46 (56)	62 (76)**
Other Lesions (in %)$$	1(1)	1 (1)	0	3 (4)	3 (4)	0
Iridocyclitis (advanced stage)	1 (1)	2 (2)	7 (9)**	2 (2)	3 (4)	8 (10)**
Iridocyclitis (early stage) (in %)	34 (42)	21 (26)	11 (13)**	33 (40)	19 (23)	11(13)**
Iridocyclitis (torpide, in evolution)(in %)	9 (11)	12 (15)	0*	9 (11)	10 (12)	0*
**Lens (Cataract)**						
Other Lesions non Cataract (in %) §§	3 (4)	3 (4)	3 (4)	5 (6)	5 (6)	4 (5)
Cataract (early stage; PES clearly visible) (%)	13 (16)	14 (17)	18 (22)	13 (16)	15 (18)	18 (22)
Cataract (in evolution; PES difficult to examine) (in %)	1 (1)	4 (5)	10 (12)**	1 (1)	4 (5)	8 (10)**
Cataract (mature; PES not visible) (in %)	2 (2)	2 (2)	9 (11)	2 (2)	0	10 (12)**
Lens Normal (in %)	63 (77)	59 (72)	42 (51)	61 (74)	58 (71)	42 (51)
**Papillitis**						
Optic Disc Atrophy (secondary) (%)	7 (8)	10 (12)	0*	7 (9)	11 (13)	0*
Other Lesions (in %)&&	1 (1)	1 (1)	12 (15)**	0	1 (1)	14 (17)**
Excavation (in %)	2 (2)	4 (5)	4 (5)	3 (4)	3 (4)	4 (5)
Impossible to examine (in %)	0	3 (4)	0	1 (1)	3 (4)	0
Papille normal (in %)	49 (60)	60 (73)&	46 (56)	49 (60)	59 (72)&	45 (55)
Papillitis (avanced stage) (%)	0	0	6 (7)	0	0	4 (5)
Papillitis (early stage) (in %)	23 (28)	4 (5)&	14 (17)**	22 (27)	5 (6)&	15 (18)**
**Chorioretinitis**						
Other Lesions (in %) §§§	1 (1)	4 (5)	5 (6)	3 (4)	4 (5)	4 (5)
Chorioretinitis (advanced) (in %)	15 (18)	14 (17)	17 (21)	18 (22)	19 (23)	20 (24)
Chorioretinitis (early stage) (%)	42 (51)	38 (46)	10 (12)**	42 (51)	35 (43)	11 (13)**
Chorion Normal (in %)	24 (30)	26 (32)	50 (61)**	19 (23)	24 (29)	47 (57)**

$ PIT =  post initial treatment.

PES =  Posterior Eye Segment.

$$ Other Lesions: Phtyse, Pupille scleroatrophic, Synechie post trauma, Pigments on posterior Capsule.

§§ Other Lesions: Phtyse, dead O.volvulus microfilariae on posterior capsule.

&& Other Lesions: optic cup/disc >0.5.

§§§ Other Lesions  =  retina detachment from the retinal pigment epithelium, drusen.

*p<0.001 compared to 4 years PIT;

**p<0.001 compared to before ivermectin;

***p<0.0001 compared to before ivermectin and 4 years PIT.

& p<0.01 compared to before ivermectin.

### Lesions of the posterior eye segment

Papillitis and chorioretinitis were present in 27%/28% (rE/lE) and 69%/73% (rE/lE) of the patients, respectively (Table 6). Until 4 years PIT, both early and advanced stage papillitis lessened to 5%/6% (rE/lE) and chorioretinitis to 63%(rE) and 66%(lE). At 23 years PIT, papillitis was present in 24%/23% (rE/lE) of the patients and chorioretinitis was observed in 33%/37% (rE/lE) of the patients; this regression is due to the fact that only in 77/78 (rE/lE) patients the fundus was accessible for examination because of anterior segment lesions. Only early-stage papillitis and chorioretinitis regressed, whilst advanced stage papillitis enhanced from 0% to 7%/5% (rE/lE) while severe chorioretinitis present in 18%/22% (rE/lE) of the patients at before treatment was found enhanced by 2–3% at 23 years PIT.

### Multivariate correlations analysis of patients, groups, treatment and ocular pathology

Cataract and visual impairment correlated positively (p<0.0001) with the patients' age and the years PIT such disclosing the age-related decline of visual functions in the patient cohort (Table 7). A negative correlation with age and the years PIT was present for MFAC, DMFC, PK (for each p<0.0001) and iridocyclitis (p = 0.0002), and this highlighted the positive effect of the repeatedly applied ivermectin treatments. The visual acuity impairment throughout the duration of study was observed to have a strong positive correlation with papillitis, chorioretinitis, iridocyclitis, SK and cataract (for each p = 0.0001 or less) such disclosing the progression of ocular pathology and visual impairment in onchocerciasis patients despite treatment. The highest positive correlations for ocular pathologies were for papillitis and chorioretinitis (r = 0.40), iridocyclitis and chorioretinitis (r = 0.38), iridocyclitis and MFAC (r = 0.37), and MFAC and DMFC (r = 0.35) (for each p<0.0001).

**Table 7:** Multivariate correlation analyses of ocular pathology and visual impairment in onchocerciasis patients treated annually with ivermectin for 23 years.

Variable	Variable	Spearman ρ	p value
Age	Cataract	0,2949	<0.0001
Age	Punctate Keratitis	−0,2934	<0.0001
Age	Visual Impairment	0,2696	<0.0001
Age	MF in the AC of the eye	−0,2461	0,0001
Years PIT	MF in the AC of the eye	−0,4103	<0.0001
Years PIT	Punctate Keratitis	−0,3425	<0.0001
Years PIT	MF in the cornea	−0,2639	<0.0001
Years PIT	Iridocyclitis	−0,2379	0,0002
Years PIT	Cataract	0,2331	0,0002
Years PIT	Visual Impairment	0,2188	0,0005
Visual Acuity	Cataract	0,5379	<0.0001
Visual Acuity	Papillitis	0,4351	<0.0001
Visual Acuity	Chorioretinitis	0,3747	<0.0001
Visual Acuity	Sclerosing Keratitis	0,2541	0,0001
Visual Acuity	Iridocyclitis	0,2511	0,0001
Cataract	Papillitis	0,2800	<0.0001
Cataract	Chorioretinitis	0,1950	0,0020
Iridocyclitis	Chorioretinitis	0,3792	<0.0001
Iridocyclitis	Cataract	0,2593	<0.0001
Iridocyclitis	Papillitis	0,2202	0,0005
Punctate Keratitis	Sclerosing Keratitis	0,2793	<0.0001
Punctate Keratitis	Chorioretinitis	0,1991	0,0016
Sclerosing Keratitis	Cataract	0,2567	<0.0001
Sclerosing Keratitis	Chorioretinitis	0,2553	<0.0001
Sclerosing Keratitis	Iridocyclitis	0,2149	0,0007
Dead MF in the cornea	Sclerosing Keratitis	0,2818	<0.0001
Dead MF in the cornea	Iridocyclitis	0,2282	0,0003
Dead MF in the cornea	Punctate Keratitis	0,2276	0,0003
MF in the AC of the eye	Iridocyclitis	0,3726	<0.0001
MF in the AC of the eye	Dead MF in the cornea	0,3511	<0.0001
MF in the AC of the eye	Sclerosing Keratitis	0,3162	<0.0001
MF in the AC of the eye	Punctate Keratitis	0,2583	<0.0001
MF in the AC of the eye	Chorioretinitis	0,2552	<0.0001
Papillitis	Chorioretinitis	0,4057	<0.0001

Significant correlations of p<0.0025 are shown. (MF = microfilariae; AC = anterior chamber; Years PIT = Years post initial treatment).

## Discussion

Repeated ivermectin treatments for more than two decades have persistently eliminated microfilaria (MF) of *O. volvulus* from the patients' anterior eye chamber and cornea. The regression of punctate keratitis, early-stage iridocyclitis and chorioretinitis and to some extent scelorosing keratitis confirm ivermectin as an effective long term control means against ocular pathology in human *O. volvulus* infection [9], [10], [19], and the efficacy of annually repeated treatments extends to disease prevention, as it may halt migration of MF of *O. volvulus* into the eye and evolution of onchocerciasis-related ocular lesions. Previous studies accomplished in Africa have similarly observed that annual treatments for 2 to 8 years strongly reduced prevalence of ocular MF [10], [20], [21], [22], [23]. In the Americas, a complete reduction of disease of the anterior eye segment has been achieved with semi annual ivermectin dosing for 14 years [24], [25]. In African onchocerciasis cohorts, ivermectin therapy applied bi-annually or annually for up to eight years lessened the number of ocular MF and significantly improved anterior eye segment lesions, despite persisting skin MF [22], [23].

Punctate keratitis (PK) lesions did not disappear or regress in our patients' cohort until 4 years PIT, and PK may persist even after 10 years of ivermectin [25]. At 23 years PIT, PK lesions were completely resolved. Such long duration for complete clearance of MF and resolution of punctuate opacities is most likely due to the low physiologic activity and turnover of corneal epithelia and endothelia. Earlier clinical studies which have followed ivermectin-treated onchocerciasis patients for several years found clear indications that early lesions of the anterior eye segment will regress including sclerosing keratitis and iridocyclitis [9], [19], [23]. In our patients, only early-stage sclerosing keratitis regressed and this occurred in half of the effected individuals only, and similarly, only early-stage iridocyclitis ameliorated significantly. Previous observations have found similar regression of iridocyclitis within shorter intervention times [10], [23], and from our observations we may conclude that advanced anterior eye segment lesions may not regress despite repeated annual ivermectin treatments for two decades. However, none of the patients presented at 23 years PIT with new or evolving iridocyclitis suggesting that inflammatory processes induced by MF or circulating filarial antigens may not have occurred.

Despite two decades of treatment with ivermectin, posterior eye segment lesion did not evolve favourably, only early-stage papillitis lesions and early-stage chorioretinitis cases were reduced by one third at 23 years PIT whilst advanced-stage papillitis and chorioretinitis cases augmented. Such unfavourable development has been described previously in onchocerciasis patients studied for 3 years [26] indicating that immune pathologic processes, i.e. destruction of cells and adjacent tissues of the retina, continued despite the ivermectin-facilitated clearance of MF from skin and eyes. Certain advanced retinal lesions may regress by secondary revascularisation [19], but such changes we could not observe. All our patients were resident in the rural Central and Kara Regions of Togo where the risk for onchocercal blindness was medium to high [27], and this area was one of the special intervention zones [19] where ongoing parasite transmission required extended vector control and intensified ivermectin distribution. The onchocerciasis control measures as applied during the past decades prevented re-emergence of patent *O. volvulus* infections in our patients and halted the dispersion and migration of MF into the anterior eye segment. An evolution of new lesions of the cornea was not observed, but already existent advanced ocular pathology did not resolve.

The degradation of visual acuity in our patients is mainly due to the persistence of advanced *O.volvulus*–induced posterior eye segment lesions and secondary anomalies, mostly the evolution of cataract - the main cause for visual impairment worldwide [28] and in sub-Saharan Africa total blindness due to cataract ranges between 21% and 67% [29]. In urban Togo, the main ocular blinding disease was also found to be cataract with 8.3% prevalence [30], while in our patients, resident in rural central and northern Togo, the prevalence and incidence of cataract was higher and rising with age. Cataract correlated positively with years' post initiation of treatment thus not supporting a causal preventive effect of ivermectin on its evolution. In Uganda, the impact of community-directed treatment with ivermectin (CDTI) on ocular manifestations showed a paucity of acute ocular onchocerciasis-related lesions but a significant presence of irreversible onchocerciasis-related ocular lesions - not only cataract but also optic atrophy and chorioretinitis [31]. In elderly members (>60 years) of a rural community in West Africa, more than 30% were with blindness and low vision, and age-related macular degeneration and glaucoma dominated next to cataract. These studies highlighted the substantial increases of blindness due to posterior eye segment anomalies and low vision in rural dwellers [32], [33]. In our cohort, none of the early-stage onchocercal posterior eye lesions became a primary cause for blindness and recent data analysis did not find evidence that ivermectin will prevent visual acuity loss [34]. Thus, in addition to the advanced non-resolving anterior and posterior eye segment onchocercal pathologies, age-related cataract and further ocular anomalies have to be considered as contributors to the observed visual deterioration in our patients' cohort.

This longitudinal cohort study disclosed that repeated ivermectin therapy will persistently eliminate MF of *O.volvulus* from the AC and cornea, achieve complete regression of PK, and advanced sclerosing keratitis will stabilize. These results endorse observations from previous studies of shorter duration [11], [20], [23], and also suggest, that the regression of certain ocular manifestations is achievable within a decade of CDTI activities. Annual ivermectin treatments for 23 years did not significantly improve existing advanced posterior eye segment lesions, and visual acuity in patient gradually lessened, and this aggravation developed together with other ocular age-related anomalies. Irreversible posterior onchocerciasis-related ocular lesions which were already present in patients before the initial ivermectin treatment, did not resolve, and this has similarly been observed in earlier investigations of shorter duration [26], [32]. The long term application of ivermectin has the potential to stop the evolution of ocular onchocerciasis, but parasite eradication in Africa may require control activities associated with a macrofilaricidal drug. The depletion of Wolbachia endosymbionts in adult *O. volvulus* by doxycycline antibiotics will accelerate the death of MF and adult *O. Volvulus* [35], and the rapid disappearance of MF from the eye anterior chamber and cornea when ivermectin is combined with doxycycline [36] may provide an additional measure for prevention and elimination of *O. volvulus* infection-associated ocular pathologies; such combined therapeutic approaches may supplement the available means for onchocerciasis elimination.

## Supporting Information

Protocol S1 **Onchocerciasis Ivermectin Study Examination Sheet Ophthalmology: 1985-1989.** Study protocol sheets (MSD-Nr519: Ivermectin (MK933) versus placebo in onchocerciasis ophthalmologic examinations (OE).
(PDF)

Checklist S1 **CONSORT 2010 checklist.** Checklist of information reporting a clinical trial.
(DOC)
