# Supplementary material for: Sustainable Control of Onchocerciasis: Ocular Pathology in Onchocerciasis Patients Treated Annually with Ivermectin for 23 Years: A Cohort Study
Source: PLoS One. 2014 Jun 2;9(6):e98411. doi: 10.1371/journal.pone.0098411 (PMC4041847; doi:10.1371/journal.pone.0098411)
Supplement: Protocol S1 — Onchocerciasis Ivermectin Study Examination Sheet Ophthalmology: 1985-1989. Study protocol sheets (MSD-Nr519: Ivermectin (MK933) versus placebo in onchocerciasis ophthalmologic examinations (OE). (PDF) [file pone.0098411.s001.pdf]

Erstbefundung
T ... Hautschnitt

IVERMECTIN (MK-933)
# VS PLACEBO IN ONCHOCERCIASIS
OPHTHALMOLOGIC EXAMINATION

OE1

IIN	STUDY NO.	PROTOCOL NO.		ALLOCATION NO.
		519		B 3

☐ PRE RETREAT. (MO.18) ☐ DAY 3 ☐ MONTH 24 ☐ MONTH 30 ☐ MONTH 36 ☐ MONTH 42 ☐ MONTH 48 ☐ MONTH 54

DATE EXAMINED 13 / 11 / 89 (day/mo/yr)

VISUAL ACUITY (6 metres; 20 feet; test with distance spectacle correction if normally used)

☒ Snellen/Illiterate E
☐ Other ______

	RE	LE
Uncorrected	5/4	5/4
Corrected/Pinhole		

PERIPHERAL VISUAL FIELDS (Confrontation method)

	RE		LE
Normal	☒	1	☒
20° – 10°	☐	2	☐
Less than 10°	☐	3	☐
Absent	☐	4	☐

Other Abnormality ☐ None, or describe below:

PUPILLARY REFLEX (Circle N = normal or A = abnormal)

(1) (2) (1) (2)
(N) A (N) A

If abnormal, describe below:
slightly slow

EXTERNAL EXAMINATION (Circle N = normal or A = abnormal)

	RE		LE	
Eyelids	(N)	A	(N)	A
Conjunctiva	N	(A)	N	(A)
Eyelid eversion	N	(A)	N	(A)
Iris, Pupil	(N)	A	(N)	A
Ocular Movement	(N)	A	(N)	A

Other Abnormality ☐ None, or describe, draw, photograph below or on Form OE5.
mild papillary conjunctivitis

Investigator's Name

CLINICAL RESEARCH – INTERNATIONAL
MERCK SHARP & DOHME RESEARCH LABORATORIES

WORK SHEET

0683GI

Printed in U.S.A.

IVERMECTIN (MK-933)
# VS PLACEBO IN ONCHOCERCIASIS
OPHTHALMOLOGIC EXAMINATION

OE 2

IIN	STUDY NO.	PROTOCOL NO.		ALLOCATION NO.
		519-19		#3

☐ PRE RETREAT. (MO.18) ☐ DAY 3 ☐ MONTH 24 ☐ MONTH 30 ☐ MONTH 36 ☐ MONTH 42 ☐ MONTH 48 ☐ MONTH 54

DATE EXAMINED 11/89 (day/mo/yr)

SLIT LAMP EXAMINATION

ANTERIOR CHAMBER (after head dependant for 5 minutes)

	RE	LE
Microfilariae (count)	0	0
Flare	0	0
Cells	0	0

Grade as: 0 = Absent, 1 = Mild, 2 = Moderate, 3 = Severe

Other Abnormality ☐ None, or describe below:

CORNEA

		RE	LE
Microfilariae counts	a) live	0	[illegible]
	b) dead	0	0
	c) locations		
Punctate Opacities	a) counts	3	0
	b) locations		

Limbitis

	RE YES	RE NO	LE YES	LE NO
Limbal vessel dilation	☒	☐	☐	☒
Limbal edema	☐	☒	☐	☒
White globular opacities	☐	☒	☐	☒

Grade as: 0 = Absent, 1 = Mild, 2 = Moderate, 3 = Severe → ____ ____

CLINICAL RESEARCH - INTERNATIONAL
MERCK SHARP & DOHME RESEARCH LABORATORIES

Investigator's Name

WORK SHEET

0683HI

Printed in U.S.A.

IVERMECTIN (MK-933)
VS PLACEBO IN ONCHOCERCIASIS
OPHTHALMOLOGIC EXAMINATION

OE 3

IIN	STUDY NO.	PROTOCOL NO.		ALLOCATION NO.
		519-19		H3

☐ PRE RETREAT. (MO.18) ☐ DAY 3 ☐ MONTH 24
☐ MONTH 30 ☐ MONTH 36 ☐ MONTH 42 ☐ MONTH 48
☐ MONTH 54

DATE EXAMINED 11/88 (day/mo/yr)

SLIT LAMP EXAMINATION (cont'd)

CORNEA (cont'd)

Sclerosing Keratitis

	RE	LE
Absent	☒	☒
Limbal Haze	☐	☐
Nasal or Temporal	☐	☐
Confluent Inferiorly	☐	☐
Pupil Covered	☐	☐

Other Abnormality ☐ None, or describe below:

______

______

LENS

	RE YES (1)	RE NO (2)	LE YES (1)	LE NO (2)
Opacity	☐	☒	☐	☒

Describe abnormality and/or location of opacity:

______

______

ANTERIOR VITREUM

	RE YES	RE NO	LE YES	LE NO
Syneresis	☐	☒	☐	☒
Opacities	☐	☒	☐	☒

Other Abnormality ☐ None, or describe below:

______

______

IRIS (Circle N = normal or A = abnormal) . . . . RE: (N) A LE: (N) A

If abnormal, describe below:

______

______

INTRAOCULAR PRESSURE (mmHg)

(Goldman Applanation Tonometer) Schiötz

	RE	LE
First Reading	15,6	15,6
Second Reading		
Third Reading		

CLINICAL RESEARCH - INTERNATIONAL
MERCK SHARP & DOHME RESEARCH LABORATORIES

Investigator's Name

WORK SHEET

068311

Printed in U.S.A.

IVERMECTIN (MK-933)
VS PLACEBO IN ONCHOCERCIASIS
OPHTHALMOLOGIC EXAMINATION

OE4

IIN	STUDY NO.	PROTOCOL NO.		ALLOCATION NO.
		51949		43

☐ PRE RETREAT. (MO.18) ☐ DAY 3 ☐ MONTH 24 ☐ MONTH 30 ☐ MONTH 36 ☐ MONTH 42 ☐ MONTH 48 ☐ MONTH 54

DATE EXAMINED 11/89 (day/mo/yr)

OPHTHALMOSCOPY

DILATED FUNDUS EXAMINATION*

	RE (1)	RE (2)	LE (1)	LE (2)
Optic Disc (circle N = normal or A = abnormal)	(N)	A	(N)	A

If abnormal, describe below:

___

	RE		LE	
Cup/Disc Ratio	___		___	
	YES	NO	YES	NO
Posterior Vitreous Detachment	☐	☐	☐	☐
Macula (circle N = normal or A = abnormal)	(N)	A	(N)	A

If abnormal, describe below:

___

Peripheral Retina

Grade as: 0 = None
1 = Mild
2 = Severe
3 = Active

	RE		LE	
Retinitis	___		___	
	YES	NO	YES	NO
Hemorrhage	☐	☑	☐	☑
Retinal Holes	☐	☑	☐	☑
Lattice Degeneration	☐	☑	☐	☑
Paving Stone Degeneration	☐	☑	☐	☑

Other Abnormality ☐ None, or describe:

___

***Note: All abnormal signs must be located on the accompanying diagrams:**

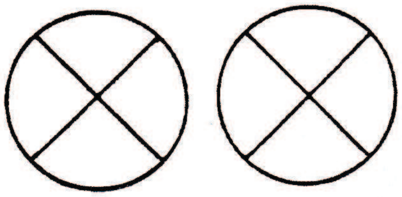

CLINICAL RESEARCH – INTERNATIONAL
MERCK SHARP & DOHME RESEARCH LABORATORIES

Investigator's Name

WORK SHEET

Printed in U.S.A.

IVERMECTIN (MK-933)
VS PLACEBO IN ONCHOCERCIASIS
OPHTHALMOLOGIC EXAMINATION

OE5

IIN	STUDY NO.	PROTOCOL NO.		ALLOCATION NO.
		519-19		

☐ PRE RETREAT. (MO.18) ☐ DAY 3 ☐ MONTH 24 DATE EXAMINED ______ (day/mo/yr)

☐ MONTH 30 ☐ MONTH 36 ☐ MONTH 42 ☐ MONTH 48 ☐ MONTH 54

FUNDUS PHOTOGRAPHY (Optional)

	RE	LE
(Circle N = normal or A = abnormal)	N A	N A

If abnormal, describe below:

______

______

FLUORESCEIN ANGIOGRAPHY (Optional)

	RE	LE
(Circle N = normal or A = abnormal)	N A	N A

If abnormal, describe below:

______

______

IVERMECTIN (MK-[illegible])
# VS PLACEBO IN ONCHOCERCIASIS
POST TREATMENT OPHTHALMOLOGIC EXAMINATION

OE 6

3

IIN	STUDY NO.	PROTOCOL NO.
[redacted]	[redacted]	519

OPHTHALMOLOGIC EXAMINATIONS — CHANGES POST TREATMENT

14/11/89

☐ DAY 3 – 4 ☐ MONTH 3 ☐ MONTH 6

NOTE TO THE INVESTIGATOR: Please review the data you have provided on Forms OE1 — OE 5. Was there a significant change from the Previous Examination? Check 'NO' or 'YES' below, and if 'YES', describe change.

OPHTHALMOLOGIC EXAMINATION	SIGNIFICANT CHANGE? NO	YES (and describe)
VISUAL ACUITY		5/5 5/5
PERIPHERAL VISUAL FIELDS	✓	
PUPILLARY REFLEX	✓	
EXTERNAL EXAMINATION	✓	
SLIT LAMP: ANTERIOR CHAMBER	✓	
CORNEA	✓	
LENS		
ANTERIOR VITREUM		
IRIS	✓	
INTRAOCULAR PRESSURE		
OPHTHALMOSCOPE: OPTIC DISC		
CUP/DISC RATIO		
POSTERIOR VITREOUS DETACHMENT		
MACULA		
PERIPHERAL RETINA		
FUNDUS PHOTOGRAPHY		
FLUORESCEIN ANGIOGRAPHY		

COMMENTS:

CLINICAL RESEARCH — INTERNATIONAL
MERCK SHARP & DOHME RESEARCH LABORATORIES

WORK SHEET

Investigator's Name
